# Protection from pulmonary ischemia-reperfusion injury by adenosine A2A receptor activation

**DOI:** 10.1186/1465-9921-10-58

**Published:** 2009-06-26

**Authors:** Ashish K Sharma, Joel Linden, Irving L Kron, Victor E Laubach

**Affiliations:** 1 Department of Surgery, University of Virginia Health System, Charlottesville, Virginia, USA; 2 Department of Medicine, University of Virginia Health System, Charlottesville, Virginia, USA

## Abstract

**Background:**

Lung ischemia-reperfusion (IR) injury leads to significant morbidity and mortality which remains a major obstacle after lung transplantation. However, the role of various subset(s) of lung cell populations in the pathogenesis of lung IR injury and the mechanisms of cellular protection remain to be elucidated. In the present study, we investigated the effects of adenosine A_2A _receptor (A_2A_AR) activation on resident lung cells after IR injury using an isolated, buffer-perfused murine lung model.

**Methods:**

To assess the protective effects of A_2A_AR activation, three groups of C57BL/6J mice were studied: a sham group (perfused for 2 hr with no ischemia), an IR group (1 hr ischemia + 1 hr reperfusion) and an IR+ATL313 group where ATL313, a specific A_2A_AR agonist, was included in the reperfusion buffer after ischemia. Lung injury parameters and pulmonary function studies were also performed after IR injury in A_2A_AR knockout mice, with or without ATL313 pretreatment. Lung function was assessed using a buffer-perfused isolated lung system. Lung injury was measured by assessing lung edema, vascular permeability, cytokine/chemokine activation and myeloperoxidase levels in the bronchoalveolar fluid.

**Results:**

After IR, lungs from C57BL/6J wild-type mice displayed significant dysfunction (increased airway resistance, pulmonary artery pressure and decreased pulmonary compliance) and significant injury (increased vascular permeability and edema). Lung injury and dysfunction after IR were significantly attenuated by ATL313 treatment. Significant induction of TNF-α, KC (CXCL1), MIP-2 (CXCL2) and RANTES (CCL5) occurred after IR which was also attenuated by ATL313 treatment. Lungs from A_2A_AR knockout mice also displayed significant dysfunction, injury and cytokine/chemokine production after IR, but ATL313 had no effect in these mice.

**Conclusion:**

Specific activation of A_2A_ARs provides potent protection against lung IR injury via attenuation of inflammation. This protection occurs in the absence of circulating blood thereby indicating a protective role of A_2A_AR activation on resident lung cells such as alveolar macrophages. Specific A_2A_AR activation may be a promising therapeutic target for the prevention or treatment of pulmonary graft dysfunction in transplant patients.

## Background

Ischemia-reperfusion (IR)-induced lung injury remains the major cause of primary graft failure after lung transplantation [1,2]. IR injury causes significant mortality and morbidity in the early post-operative period and is reported to be an independent predictive factor for the development and progression of bronchiolitis obliterans syndrome, which is the most common cause of death after lung transplantation [1,3]. We have previously demonstrated that alveolar macrophage activation [4] and alveolar type II epithelial cell activation [5] are associated with the induction of lung IR injury. An event which follows macrophage and epithelial cell activation is neutrophil activation and infiltration into lung tissue which results in severe pulmonary dysfunction in the early post-transplant period [6-8]. Pulmonary IR injury also entails the induction of pro-inflammatory cytokines and chemokines [9,10], and the contribution of TNF-α, IL-1β, IL-6 and KC (CXCL1) in the genesis and progression of lung IR injury has been demonstrated [5,11,12].

One major anti-inflammatory mechanism after lung injury is mediated by the release of adenosine [13]. Adenosine receptors are found on various cell types, and the activation of these receptors often results in suppression of inflammatory function [14-17]. The A_2A _adenosine receptor (A_2A_AR) is one of four subtypes of the G protein-coupled adenosine receptor family which includes A_1_, A_2A_, A_2B _and A_3_. Adenosine receptor sub-classification has shown specifically that activation of A_2A_AR produces anti-inflammatory responses and prevents leukocyte adhesion [18,19]. Recent studies have shown that pharmacologic activation of A_2A_AR restores functional integrity in renal, cardiac, hepatic and spinal cord IR injury models [20-25]. A_2A_AR activation during reperfusion has also been shown to ameliorate lung IR injury while decreasing cellular and molecular inflammatory markers [26,27]. A_2A_ARs are predominantly expressed on inflammatory cells including neutrophils, mast cells, macrophages, T cells, monocytes and platelets [28,29]. The attenuation of IR injury by A_2A_AR activation is postulated to involve a purinergic regulatory process whereby the A_2A_AR coupled to a stimulatory G protein leads to an increase in cyclic adenosine monophosphate (cAMP), thereby resulting in reduced cytokine release and inactivation of inflammatory cells [30,31].

This study focuses on the role of resident lung leukocytes in IR injury and the effects of A_2A_AR activation on these cells using an isolated, buffer-perfused mouse model of lung IR injury. This model allows the investigation of specific and direct effects of A_2A_AR activation on lung function independent of circulating platelets and neutrophils. The anti-inflammatory actions of selective A_2A_AR agonists have been attributed to circulating leukocytes in previous studies. However, in the present study, we hypothesize that specific activation of A_2A_AR on resident lung cells would attenuate pulmonary injury and dysfunction after IR despite the absence of circulating platelets and leukocytes from blood reperfusion.

## Methods

### Animals and study design

We utilized 8–10 week old wild-type (WT) C57BL/6J mice (The Jackson Laboratory, Bar Harbor, ME) and A_2A_AR knockout (KO) mice congenic to C57BL/6J [32]. Three groups of animals were studied (n = 6/group); a sham group, an IR group and an IR+ATL313 group. Lungs in the IR group were subjected to 1 hr ischemia followed by 1 hr reperfusion with Krebs-Henseleit buffer. As a control, Sham lungs received 2 hr reperfusion without ischemia, and the final 1 hr of perfusion in the sham lungs were compared with the 1 hr of reperfusion in the IR group. The IR+ATL313 group was identical to the IR group except that ATL313 (30 nM) was added to the perfusate buffer at the beginning of the reperfusion period. ATL313 (a gift from Adenosine Therapeutics, LLC, Charlottesville, VA) is a potent and highly specific activator of A_2A_AR [32]. The dose of ATL313 used in this study (30 nM) had no significant effects on pulmonary function or hemodynamics in lungs from sham animals (data not shown). A_2A_AR KO mice were also subjected to IR and IR+ATL313 as described above. This study was conducted under protocols approved by the University of Virginia's Institutional Animal Care and Use Committee. All animals received humane care in compliance with the "Principles of Laboratory Animal Care" formulated by the National Society for Medical Research, and "The Guide for the Care and Use of Laboratory Animals", prepared by the National Academy of Science and published by the National Institutes of Health.

### Isolated, buffer-perfused lung IR model

For this study, we used an isolated, buffer-perfused mouse lung system (Hugo Sachs Elektronik, March-Huggstetten, Germany) as previously described by our laboratory [4]. Mice were anesthetized with ketamine and xylazine. A tracheotomy was performed, and animals were ventilated with room air at 100 breaths/min at a tidal volume of 7 μl/g body weight with a positive end expiratory pressure of 2 cm H_2_O using the MINIVENT mouse ventilator (Hugo Sachs Elektronik, March-Huggstetten, Germany). A midline abdominal incision was made, and the inferior vena cava was cannulated with a 30-gauge needle and injected with 500 units of heparin. Animals were exsanguinated by inferior vena caval transection. The subdiaphragmatic portion of the animal was excised and discarded. The anterior chest plate was removed, exposing the lungs and heart. A 4-0 silk suture was passed behind the pulmonary artery (PA) and the aortic root. A partial half-knot was created with the suture, leaving room for the cannula to be passed into the PA. A small curvilinear incision was made in the right ventricular outflow tract with the perfusate flowing at 0.2 ml/min, and the PA cannula was passed through the pulmonary valve into the PA. The partial half-knot was then tightened. The left ventricle was immediately vented with a small incision at the apex of the heart. The mitral apparatus was carefully dilated and the left atrial cannula was passed through the mitral valve into the left atrium. The placement of the left atrial and the PA cannulas were further confirmed by pressure tracings generated by the PULMODYN data acquisition system (Hugo Sachs Elektronik). The lungs were then perfused at a constant flow of 60 μl/g body wt/min with Krebs-Henseleit buffer (Sigma-Aldrich, St. Louis, MO) containing 0.1% glucose and 0.3% HEPES (335–340 mOsmol/kg H_2_O). The Krebs solution was prepared to mimic mixed venous blood using an oxygenator (Living Systems Instrumentation, Burlington, VT) with titrated gases generating a pH = 7.35–7.40, a pO_2 _= 60–70 mmHg, and a pCO_2 _= 50–60 mmHg. The buffered perfusate and isolated lungs were maintained at 37°C throughout the experiment by use of a circulating water bath.

Isolated lungs were allowed to equilibrate on the apparatus during a 15-min stabilization period. After equilibration, ventilation was decreased to 50 breaths/min, and the fraction of inspired oxygen was decreased to <1%. To initiate the ischemic period, hypoxic ventilation was maintained with 95% nitrogen and 5% carbon dioxide, and perfusion was arrested. After 60 min of ischemia and hypoxic ventilation, perfusion and room air ventilation were then resumed to initiate the reperfusion period. Hemodynamic and pulmonary function parameters were continuously recorded throughout the reperfusion period by the PULMODYN data acquisition system (Hugo Sachs Elektronik). Ventilation with hypoxic gas rather than stopping ventilation altogether during ischemia was performed to avoid atelectasis while still maintaining ischemia. Atelectasis and reexpansion has been shown to induce injury involving edema, free radical generation, and apoptosis [33,34]. This reexpansion-induced injury could obscure the effects of IR injury, an issue we wished to avoid.

### Bronchoalveolar lavage (BAL) fluid collection

After perfusion, lungs were lavaged with 0.5 ml saline via tracheotomy. This procedure was performed three times, and the fluid was pooled together. An average of 1.2 ml total BAL fluid was collected from each mouse. BAL fluid was centrifuged at 4°C (1500 *g *for 15 min), and the supernatant was stored at -80°C.

### Cytokine and chemokine protein analysis

Cytokine and chemokine protein content in BAL fluid was quantified using the Bioplex Bead Array technique using a mouse-specific multiplex cytokine panel assay (Bio-Rad Laboratories, Hercules, CA). The microplates were analyzed by the Bioplex array reader which is a fluorescent-based flow cytometer employing a specific bead-based multiplex technology, each of which is conjugated with a reactant specific for a different target cytokine. The array reader quantifies the magnitude of the bead fluorescence intensity associated with each target protein.

### Lung wet/dry weight ratio

Lung wet/dry weight ratio was used as an indicator of pulmonary edema using separate groups of animals (n = 5/group). The lower lobe of the right lung from each animal was harvested, weighed and placed in a vacuum oven (at 54°C) until a stable, dry weight was achieved. The ratio of lung wet weight to dry weight was then calculated.

### Vascular permeability assay

As another indicator of lung injury, lung vascular permeability was assessed using separate groups of animals (n = 5/group). At the completion of reperfusion, the perfusion buffer (Krebs Henseleit solution) was replaced with 30 mg/ml bovine serum albumin (BSA) solution (in PBS), and the lungs were perfused for an additional 5 min at the same flow rate as during reperfusion. After this, the BSA solution was changed back to Krebs Henseleit solution, and perfusion was continued for an additional 5 min to wash out the BSA solution from the lung vasculature. Using a BSA ELISA kit (Immunology Consultants Laboratory, OR), BSA concentration in BAL fluid was measured according to the manufacturer's instructions.

### Myeloperoxidase (MPO) measurement

MPO, which is expressed in neutrophils, was measured in BAL fluid as an indicator of neutrophil infiltration into alveolar spaces. An MPO ELISA kit (Cell Sciences, Canton, MA) was utilized as instructed by the manufacturer.

### Statistical analysis

Values are presented as the mean ± standard error of the mean (SEM). Analysis of variance (ANOVA) was used to determine if significant differences existed between groups. Bonferroni's HSD multiple comparison test was used to determine which groups were significantly different when the ANOVA results were significant. Data was considered significant when *p *< 0.05.

## Results

### Lung function after IR

**Figure 1:**
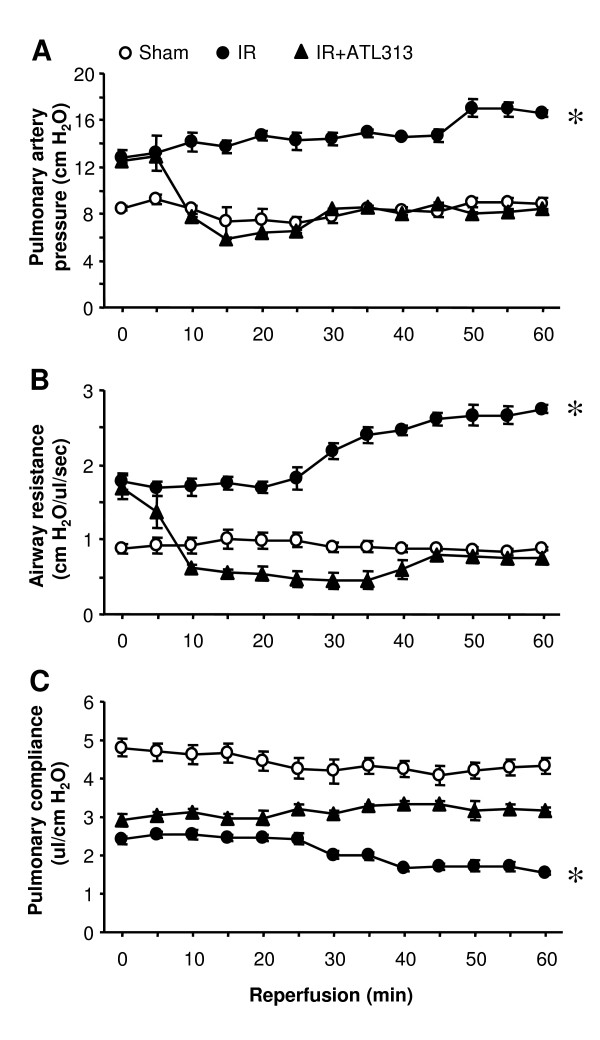
**Temporal changes in pulmonary function during reperfusion**. Pulmonary artery pressure (A), airway resistance (B), and pulmonary compliance (C) were measured throughout 60 min reperfusion in WT sham and IR lungs. Lung function is significantly impaired after IR compared to sham, and ATL313 significantly attenuated lung dysfunction. Open circles, Sham lungs undergoing perfusion only; filled circles, IR lungs reperfused after 60 min ischemia; filled triangles, IR lungs treated with ATL313 (30 nM) during reperfusion. *p < 0.01 IR vs. all.

Using the isolated, buffer-perfused mouse model of IR, lung function in WT mice was measured during the 1 hr reperfusion period after ischemia and compared to Sham lungs. Significant lung dysfunction occurred after IR as shown in Figure 1. At the end of reperfusion, IR lungs exhibited significantly increased pulmonary artery pressure (16.5 ± 0.26 vs. 8.78 ± 0.56 cm H_2_O), increased airway resistance (2.74 ± 0.05 vs. 0.87 ± 0.02 cm H_2_O/μl/sec) and reduced pulmonary compliance (1.54 ± 0.05 vs. 4.34 ± 0.19 μl/cm H_2_O) compared to Sham (p < 0.01).

### Lung dysfunction after IR is attenuated by A_2A_AR activation

To investigate the effects of A_2A_AR activation on lungs undergoing IR injury, ATL313, a specific A_2A_AR agonist, was administered during reperfusion. A significant improvement in lung function was observed in the ATL313-treated lungs compared to lungs undergoing IR alone (Figure 1). At the end of reperfusion, ATL313 significantly reduced pulmonary artery pressure (8.37 ± 0.55 vs. 16.5 ± 0.26 cm H_2_O), reduced airway resistance (0.75 ± 0.09 vs. 2.74 ± 0.05 cm H_2_O/μl/sec) and increased pulmonary compliance (3.18 ± 0.09 vs. 1.54 ± 0.05 μl/cm H_2_O) compared to IR alone (p < 0.01). In fact, lung function after IR in ATL313-treated lungs was comparable to Sham lungs.

### ATL313 specifically acts on A_2A_AR

**Figure 2:**
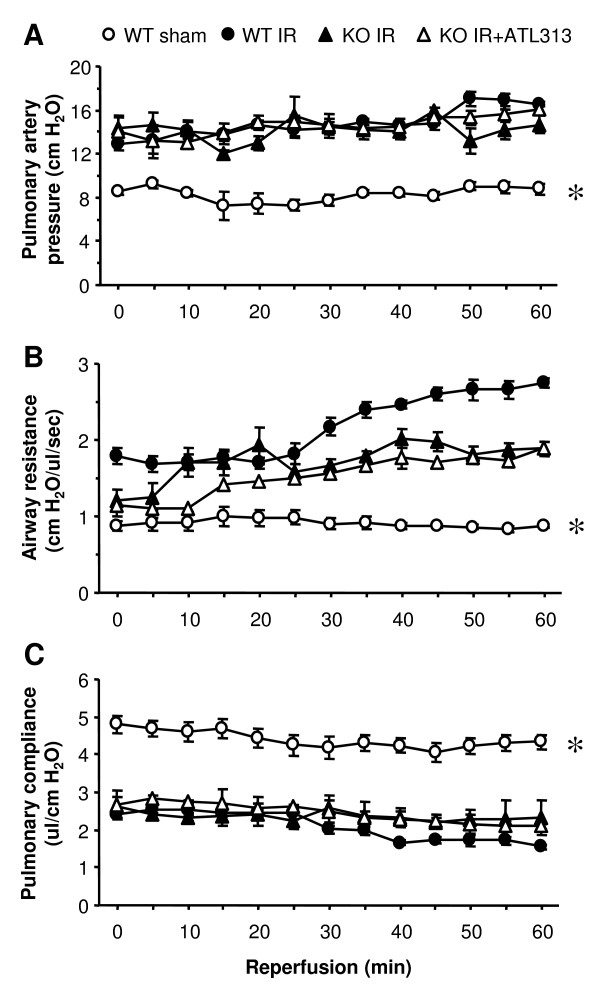
**Pulmonary function during reperfusion in A_2A_AR KO mice**. Pulmonary artery pressure (A), airway resistance (B), and pulmonary compliance (C) were measured throughout 60 min reperfusion in A_2A_AR KO lungs after IR with or without administration of ATL313. Lung function was significantly impaired in A_2A_AR KO mice, and ATL313 treatment offered no protection. Open circles, WT Sham lungs undergoing perfusion only; filled circles, WT IR lungs reperfused after 60 min ischemia; filled triangles, A_2A_AR KO IR lungs reperfused after 60 min ischemia; open triangles, A_2A_AR KO IR lungs treated with ATL313 (30 nM) during reperfusion. *p < 0.01 WT Sham vs. all.

To eliminate the possibility that ATL313 could have effects secondary to A_2A_AR activation, lung function was measured after IR in A_2A_AR KO mice with or without treatment with ATL313. Lung function was not different between sham A_2A_AR KO mice and sham WT mice (data not shown). Similar to WT mice, significant dysfunction occurred in lungs from A_2A_AR KO mice after IR (Figure 2). At the end of reperfusion, lungs from A_2A_AR KO mice displayed significantly increased pulmonary artery pressure (14.7 ± 0.83 vs. 8.78 ± 0.56 cm H_2_O), increased airway resistance (1.89 ± 0.09 vs. 0.87 ± 0.02 cm H_2_O/μl/sec) and decreased pulmonary compliance (2.32 ± 0.09 vs. 4.34 ± 0.19 μl/cm H_2_O) versus WT Sham (p < 0.01). Furthermore, treatment of A_2A_AR KO mice with ATL313 did not result in any improvement in lung function, and these mice displayed no significant difference in lung function compared to A_2A_AR KO mice undergoing IR alone (Figure 2).

### A_2A_AR activation inhibits lung IR injury

**Figure 3:**
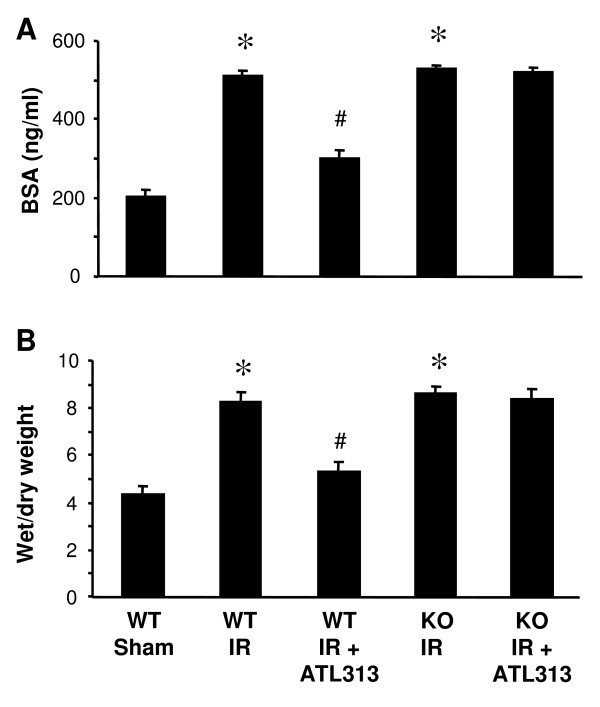
**Lung IR injury is attenuated by A_2A_AR activation**. Lung vascular permeability was assessed by measuring BSA concentration in BAL fluid (A). Lung edema was assessed by measuring wet/dry weight ratio (B). Significant lung injury (increased vascular permeability and edema) occurred after IR in WT mice which was attenuated by ATL313 treatment. Significant lung injury also occurred in A_2A_AR KO mice after IR, but ATL313 had no affect on A_2A_AR KO lungs. WT Sham, WT lungs undergoing perfusion only; WT IR, WT lungs undergoing IR; KO IR, A_2A_AR KO lungs undergoing IR; KO IR+ATL313, A_2A_AR KO IR lungs treated with ATL313 (30 nM) during reperfusion. *p < 0.001 IR vs. WT Sham; ^#^p < 0.01 WT IR+ATL313 vs. WT IR.

To assess lung injury after IR, vascular permeability (as measured by BSA content in BAL fluid) and pulmonary edema (as measured by wet/dry weight) were assessed at the end of the reperfusion period. A significant increase in vascular permeability (Figure 3A) and pulmonary edema (Figure 3B) occurred after IR in lungs from WT and A_2A_AR KO mice versus sham (p < 0.001). ATL313 significantly decreased vascular permeability and pulmonary edema in WT mice after IR (p < 0.01) but had no effect on lungs from A_2A_AR KO mice after IR (Figure 3).

### A_2A_AR activation attenuates cytokine/chemokine expression

**Figure 4:**
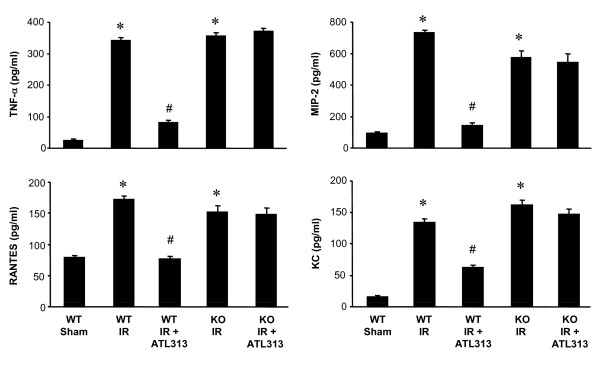
**Cytokine/chemokine expression after lung IR**. The expression of TNF-α, MIP-2 (CXCL2), RANTES (CCL5), and KC (CXCL1) in BAL fluid were significantly induced after IR in WT and A_2A_AR KO mice (*p < 0.01 vs. WT Sham). Cytokine/chemokine induction was significantly impaired by ATL313 treatment in WT mice but not in A_2A_AR KO mice (^#^p < 0.001, WT IR+ATL313 vs. WT IR). WT Sham, WT lungs undergoing perfusion only; WT IR, WT lungs undergoing IR; KO IR, A_2A_AR KO lungs undergoing IR; KO IR+ATL313, A_2A_AR KO IR lungs treated with ATL313 (30 nM) during reperfusion.

Pro-inflammatory cytokine/chemokine expression was measured in BAL fluid of WT and A_2A_AR KO mouse lungs after IR, with or without ATL313 treatment. A significant increase in the production of TNF-α, KC (CXCL1), MIP-2 (CXCL2) and RANTES (CCL5) occurred in both WT and A_2A_AR KO mice after IR (Figure 4, p < 0.01). Treatment with ATL313 significantly attenuated cytokine/chemokine activation after IR in WT mice (p < 0.001) but had no significant effect in A_2A_AR KO mice (Figure 4). In addition, there was no significant production of IL-6, MCP-1 or IFN-γ in WT or A_2A_AR KO mouse lungs after IR (data not shown).

### Neutrophil infiltration after lung IR is attenuated by A_2A_AR activation

**Figure 5:**
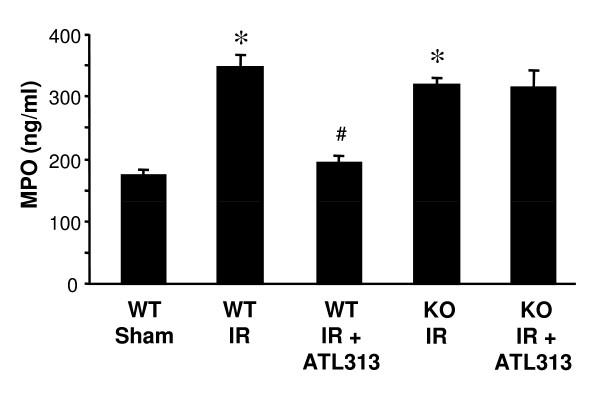
**Lung IR-induced alveolar MPO content is attenuated by A2AAR activation**. MPO content in BAL fluid was significantly induced in lungs from WT and A_2A_AR KO mice after IR (*p < 0.01 vs. WT Sham). MPO content was significantly attenuated by ATL313 treatment in WT mice but not in A_2A_AR KO mice (^#^p < 0.001, WT IR+ATL313 vs. WT IR). WT Sham, WT lungs undergoing perfusion only; WT IR, WT lungs undergoing IR; KO IR, A_2A_AR KO lungs undergoing IR; KO IR+ATL313, A_2A_AR KO IR lungs treated with ATL313 (30 nM) during reperfusion.

MPO is abundantly present in azurophilic granules of polymorphonuclear neutrophils and its increased concentration in BAL fluid is an indicator of neutrophil activation and migration into alveolar airspaces. MPO content in BAL fluid was evaluated in WT and A_2A_AR KO lungs after IR with or without ATL313 treatment. MPO content was significantly increased in WT lungs after IR compared to Sham (Figure 5, p < 0.01). Treatment with ATL313 significantly reduced MPO content in WT lungs after IR (p < 0.001). In A_2A_AR KO mice, IR also resulted in significantly increased MPO content, however ATL313 treatment did not affect MPO content in these mice (Figure 5).

## Discussion

In this study, we investigated the role of A_2A_AR activation in mediating protection from lung IR injury utilizing a buffer-perfused mouse lung model. ATL313-mediated protection was illustrated by significant reductions in mean pulmonary artery pressure and airway resistance, and significant improvement in pulmonary compliance throughout the reperfusion period following ischemia. Lungs undergoing IR also displayed increased vascular permeability and pulmonary edema which were significantly attenuated by ATL313 treatment. We cannot discount the possibility that the attenuation in vascular permeability by ATL313 may be in part due to a decrease in intravascular pressure as well as to changes in endothelial permeability. In addition, A_2A_AR KO mice demonstrated significant lung injury and dysfunction, but ATL313 treatment failed to protect these lungs after IR, indicating the specificity of ATL313 for A_2A_AR. The induction of specific pro-inflammatory cytokines/chemokines (TNF-α, KC, MIP-2 and RANTES) and increased MPO content after IR were also significantly attenuated by A_2A_AR activation in WT lungs.

The results presented in this study support other reports of protection from IR injury by specific A_2A_AR agonists [16,22,27,35,36]. More importantly, while prior studies indicate that circulating cells such as platelets and neutrophils play a significant role in the anti-inflammatory effects of A_2A_AR activation, the present study describes A_2A_AR-mediated protection in the absence of circulating cells. These results suggest that the cellular targets for ATL313-mediated protection in buffer-perfused lungs are likely resident lung leukocyte populations including alveolar macrophages, neutrophils (including marginated neutrophils) or T cells. The source of the infiltrating neutrophils in alveolar airspaces after IR must be either interstitial or marginated neutrophils in this model. We have previously shown that a significant number of marginated neutrophils are retained in buffer-perfused lungs [4]. However, the contribution of other cells, such as epithelial or endothelial cells, to A_2A_AR-mediated protection cannot be eliminated. This study suggests that the specific activation of A_2A_AR improves pulmonary injury and function after IR in a non-blood perfused system, thereby indicating the importance of resident pulmonary cells in lung IR injury.

This study highlights an emerging therapeutic role for the use of A_2A_AR agonists, such as ATL313, as a logical extension of many intrinsic defense mechanisms inasmuch as adenosine is known to accumulate in response to inflammation or IR injury. Whereas the exact mechanisms of protection are complex and poorly understood, treatment with A_2A_AR agonists has been associated with inhibition of inflammatory cytokine release, reduction of apoptotic injury, and diminution of free radical production [37-39]. In this study, the markedly significant protection offered by ATL313 in WT mice suggests an important protective role of A_2A_ARs in lung IR injury, and that specific activation of A_2A_AR, and not A_1_AR, A_2B_AR or A_3_AR, has broad anti-inflammatory effects that attenuate lung IR injury.

Although we anticipated that lung injury and dysfunction after IR would be worse in A_2A_AR KO mice, injury and function in A_2A_AR KO mice were comparable to WT mice. One possible explanation for this might be that the buffer-perfused model of IR injury, or the timeframe utilized (1 hr ischemia and 1 hr reperfusion), does not entail significant production of adenosine to result in enhanced anti-inflammatory effects in WT mice. A second possible explanation is that this model does not entail blood-perfusion whereby enhanced platelet activation and infiltration of circulating leukocytes may occur in A_2A_AR KO mice to exacerbate injury. However, using a blood-perfused mouse lung IR model involving left lung hilar clamp (1 hr ischemia followed by 2 hr reperfusion), we observed a similar pattern of injury in A_2A_AR KO mice as compared to WT mice (data not shown). A third possible explanation, and probably the most likely explanation, is that the A_2A_AR does not normally play a significant anti-inflammatory role during acute lung IR injury. It is plausible that activation of other adenosine receptors (A_1_, A_2B _and A_3_), which are all expressed in the lung [40], may have significant, anti-inflammatory roles in the pathogenesis of IR. For example, it is well documented that A_1_AR plays a role in protecting the heart [41] and kidney [42] against IR injury. However, A_1_AR antagonism has been shown to be beneficial in attenuating lung IR injury [43], results which add to the confusion regarding the role of A_1_AR in lung injury. A recent study showed that exposure to endotoxin results in augmented pro-inflammatory cytokine levels and increased leukocyte adhesion in A_2B_AR KO mice [44]. A study by Rivo et al. showed that activation of A_3_AR may protect the lung against IR injury [45]. Although it is possible that enhanced expression of other adenosine receptors could compensate for the lack of A_2A_AR in the KO mice, our data suggests that endogenous activation of A_2A_AR may not play a significant role in IR injury. The contribution of other adenosine receptors in the modulation of lung IR injury remains a subject for further investigation.

Previous studies from our group and others have demonstrated a significant role of alveolar macrophages in the initiation of lung IR injury [4,46,47]. We have also demonstrated that the intercellular interactions between alveolar macrophages and type II epithelial cells are important events in lung IR injury via activation of specific cytokines/chemokines such as TNF-α and KC, thereby leading to enhanced neutrophil chemotaxis [5]. The specific activation of A_2A_ARs on macrophages has been shown to attenuate IR injury in other organ systems such as kidney, liver and heart [21,23,25,48]. However, the role of A_2A_AR activation specifically on resident lung cell populations after IR has not been previously explored. The pro-inflammatory cascade in lung IR injury involving TNF-α has also been previously documented by our group and others [12,49,50]. These findings highlight a complex cross-talk between various lung cell populations including alveolar macrophages, type II epithelial cells and neutrophils in the progression of IR injury. Our findings suggest that cytokines and chemokines, such as TNF-α, KC and MIP-2, released by alveolar macrophages and type II epithelial cells activate neutrophils, which serve as end effecters of lung IR injury. It is known that neutrophils progressively infiltrate the transplanted lung after reperfusion and contribute to injury by releasing oxygen free radicals [2]. We also observed that increased alveolar MPO content after IR was significantly attenuated by ATL313, indicative of the protective role of A_2A_ARs on resident lung neutrophils. We have previously demonstrated the presence of marginated neutrophils in buffer-perfused mouse lungs [4]. A_2A_ARs on these marginated neutrophils, in conjunction with resident lung leukocytes such as alveolar macrophages are likely key targets for ATL313-mediated protection against IR-induced lung injury.

This study focused on the acute, anti-inflammatory effects of A_2A_AR activation. It is plausible, however, that A_2A_AR-mediated protection is also a prolonged phenomenon attenuating the chronic fibroproliferative phase of lung IR injury. However, due to the inherent limitations of the acute *ex-vivo*, buffer-perfused model, the study of long term A_2A_AR-mediated protection is beyond the scope of the present study. Another limitation of the buffer-perfused IR model is the use of hypoxic ventilation to induce ischemia instead of clamping the lung in an inflated state, which is utilized in the human lung transplantation. However, the hypoxic ventilation was used to prevent atelectasis in the buffer-perfused IR model. Moreover, the *in vivo *hilar clamp IR model entails circulating blood and thus does not specifically address the role of resident lung cells in lung IR injury, which was the focus of this study.

## Conclusion

In summary, our data suggests that attenuation of the inflammatory cascade by ATL313 activation of A_2A_ARs on resident lung cells is sufficient in preventing lung injury and dysfunction after IR. Pulmonary IR injury entails complex signaling mechanisms involving the intercellular interactions of various lung cell populations via a pro-inflammatory cascade. The selective activation of A_2A_ARs on resident lung leukocyte populations such as alveolar macrophages, marginated neutrophils, and possibly T lymphocytes by ATL313 is an anti-inflammatory intervention that inhibits neutrophil activation and subsequent infiltration and tissue injury. Thus, specific A_2A_AR activation may be a promising therapeutic target for the prevention or treatment of pulmonary graft dysfunction in transplant patients.

## Competing interests

JL and ILK were shareholders in Adenosine Therapeutics, LLC, the corporation that provided ATL313, during the time of this study.

## Authors' contributions

AKS conducted the research experiments, performed statistical analysis, and drafted the manuscript. JL and ILK helped with the analysis and interpretation of the data. VEL helped in conception and design of the experiments, analysis and interpretation of the data and drafting of the article. All authors read and approved the final manuscript.
